# 

**Published:** 1899-07-01

**Authors:** 


**Notices of Births, Deaths, and Marriages** are inserted in this column at a charge of 2s. 6d. for an announcement not exceeding four lines.

**MARRIAGE.**

Large—Hicks.—On the 22nd inst., at St. Paul's Church, Hull, by the Rev. Robert Large, M.A., assisted by the Rev. G. F. Tamplin, M.A., Vicar, Robert Emmott Large, of Flintham Lodge, Oaksey, Wilts, to K. Philippa Hicks, late Lady Superintendent of the Nurses' Co-operation, 8, New Cavendish Street, London, and youngest daughter of the Rev. Robert Hicks, late Rector of Kirk Smeaton, Yorks. (95)

### NOTICE TO CORRESPONDENTS.

All MS., Letters, Books for Review, and other matters intended for the Editor should be addressed The Editor, "The Hospital," The Hospital Building, 28 & 29, Southampton Street, Strand, W.C.

The Editor cannot undertake to return rejected MS., even when accompanied by stamped directed envelope.

**Notice to Advertisers and Subscribers.**

All Advertisements, Orders for Copies of The Hospital, and Business Communications should be addressed to **THE MANAGER (Not to the Editor),** The Hospital, The Hospital Building, 28 & 29, Southampton Street, Strand, London, W.C.

The Cost of Subscription, post free, is as follows:—Per week, 2½d.; per quarter, 2s. 8d.; per half-year, 5s. 3d.; per annum, 10s. 6d. Foreign:—Per annum, 15s. 2d., payable, in all cases, in advance.

**NOTICE.—Vol. XXV. of "The Hospital" (October, 1898, to March, 1899), in handsome cloth binding, is now on sale at the Office of this Journal, price 7s. 6d. Cases for binding the Numbers contained in this Volume 1s. 6d. Index to Vol. XXV., 2½d., post free.**

**The Monthly Part for July is now ready. Price 6d., by post 8d.**

### Scraps and Gleanings.

The cost and equipment of the new buildings of the Birmingham Homœopathic Hospital will be about £6,000.

The total amount of this year's house to house collection in connection with the Aberdeen Hospital Saturday Fund is upwards of £500.

This year's Hospital Saturday demonstration at Lymington resulted in the raising of a sum of £106 odd, which is an increase of £17 over last year's collection.

The plans of Mr. Henman for the new Royal Victoria Hospital at Belfast have been approved of. They provide for about 300 beds in 19 wards. All the wards and rooms for patients are on one floor level.

The new Infectious Diseases Hospital for Burnley is to contain 30 beds to commence with, but it is thought that it will be necessary to increase that accommodation to 104 beds, which could be done by the addition of pavilions.

At the annual meeting of the governors of the Passmore Edwards District Cottage Hospital, Tilbury, it was resolved that the committee should consider and report to a special general meeting of governors on the question of enlarging the hospital.

